# Glucosamine prevents *in vitro *collagen degradation in chondrocytes by inhibiting advanced lipoxidation reactions and protein oxidation

**DOI:** 10.1186/ar2274

**Published:** 2007-08-08

**Authors:** Moti L Tiku, Haritha Narla, Mohit Jain, Praveen Yalamanchili

**Affiliations:** 1 Department of Medicine, University of Medicine and Dentistry of New Jersey, Robert Wood Johnson Medical School, One Robert Wood Johnson Place, New Brunswick, NJ 08903, USA

## Abstract

Osteoarthritis (OA) affects a large segment of the aging population and is a major cause of pain and disability. At present, there is no specific treatment available to prevent or retard the cartilage destruction that occurs in OA. Recently, glucosamine sulfate has received attention as a putative agent that may retard cartilage degradation in OA. The precise mechanism of action of glucosamine is not known. We investigated the effect of glucosamine in an *in vitro *model of cartilage collagen degradation in which collagen degradation induced by activated chondrocytes is mediated by lipid peroxidation reaction. Lipid peroxidation in chondrocytes was measured by conjugated diene formation. Protein oxidation and aldehydic adduct formation were studied by immunoblot assays. Antioxidant effect of glucosamine was also tested on malondialdehyde (thiobarbituric acid-reactive substances [TBARS]) formation on purified lipoprotein oxidation for comparison. Glucosamine sulfate and glucosamine hydrochloride in millimolar (0.1 to 50) concentrations specifically and significantly inhibited collagen degradation induced by calcium ionophore-activated chondrocytes. Glucosamine hydrochloride did not inhibit lipid peroxidation reaction in either activated chondrocytes or in copper-induced oxidation of purified lipoproteins as measured by conjugated diene formation. Glucosamine hydrochloride, in a dose-dependent manner, inhibited malondialdehyde (TBARS) formation by oxidized lipoproteins. Moreover, we show that glucosamine hydrochloride prevents lipoprotein protein oxidation and inhibits malondialdehyde adduct formation in chondrocyte cell matrix, suggesting that it inhibits advanced lipoxidation reactions. Together, the data suggest that the mechanism of decreasing collagen degradation in this *in vitro *model system by glucosamine may be mediated by the inhibition of advanced lipoxidation reaction, preventing the oxidation and loss of collagen matrix from labeled chondrocyte matrix. Further studies are needed to relate these *in vitro *findings to the retardation of cartilage degradation reported in OA trials investigating glucosamine.

## Introduction

Osteoarthritis (OA) is characterized by the progressive degradation and loss of articular cartilage [1]. OA is the most common arthritic disease and its incidence increases with age. As population demographics changes to include more elderly individuals, this disease will have a serious impact in multiple ways. Along with the cost for health care and lost work time, individuals with OA suffer from pain and disability [2]. Currently, there is no specific treatment to prevent or retard the cartilage degradation in OA. Present treatments used for OA provide only symptomatic relief from the pain. Glucosamine sulfate, which has received attention as a putative agent that may retard cartilage structural degradation in OA, has been investigated in several OA trials [3-5]. The result on applicability of glucosamine in the clinical setting is still controversial [6-8]. Glucosamine in its various salt formulations with or without chondroitin sulfate is available over-the-counter as a nutritional supplement and is consumed by large numbers of osteoarthritic patients.

The mechanism of retardation of cartilage degradation by glucosamine is not known. Glucosamine has been shown to have a number of effects in *in vitro *chondrocyte and explant cultures [9-13]. These effects include stimulation of proteoglycan synthesis, inhibition of the degradation of proteoglycans, and inhibition of matrix metalloproteinase-3 synthesis [14-16]. Glucosamine inhibits aggrecanase activity via suppression of glycosylphosphatidylinositol-linked proteins [17]. Furthermore, glucosamine has been shown to inhibit cytokine (interleukin-1 [IL-1])-induced activation of chondrocytes and nuclear factor-kappa-B activity and to upregulate type II IL-l decoy receptor [18,19]. *In vivo*, glucosamine helps enhance healing of cartilage injury [20-23]. Glucosamine has been demonstrated to have immunosuppressive and tumor-inhibiting activity [24,25]. All these pleiotropic effects of glucosamine may individually or collectively have a chondroprotective effect.

Does the ability of glucosamine sulfate to retard cartilage structural degradation observed in OA clinical studies [3-5] involve the protection of collagen degradation? We tested the effect of glucosamine in an *in vitro *model of chondrocyte-dependent collagen degradation [26] in which collagen degradation is mediated mostly by the activation of chondrocyte lipid peroxidation resulting in aldehydic oxidation and fragmentation of cartilage collagen.

## Materials and methods

### Reagents

Calcium ionophore A23187, vitamin E, butylated hydroxytoluene, tetramethoxypropane, glucose oxidase, glucosamine hydrochloride (interchangeably described as glucosamine), and other reagents were purchased from Sigma-Aldrich (St. Louis, MO, USA). Rotta Research Laboratorium (Monza, Italy) provided glucosamine sulfate. Hydrogen peroxide of reagent grade was obtained from Fisher Scientific (part of Thermo Fisher Scientific Inc., Waltham, MA, USA). Dulbecco's modified Eagle's medium (DMEM), fetal bovine serum (FBS), Hanks' balanced salt solution (HBSS), Earl's balanced salt solution (EBSS), L-glutamine, gentamicin, HEPES buffer, penicillin, and streptomycin were purchased from Gibco-BRL (now part of Invitrogen Corporation, Carlsbad, CA, USA). Proline, L [2,3,4,5-H] with specific activity of 90 curies per millimole was obtained from American Radiolabeled Chemicals, Inc. (St. Louis, MO, USA).

### Isolation of rabbit articular chondrocytes

NZW rabbits (2.2 to 2.9 kg) of either gender were killed by intravenous injection of Beuthanasia-D special (Schering-Plough Corporation, Kenilworth, NJ, USA). The chondrocytes were isolated as described previously [26]. The viability of chondrocytes was confirmed by trypan blue exclusion. Primary chondrocytes were suspended in 10% FBS in DMEM containing antibiotics (1%) and HEPES buffer (10 mM, pH 7.4) (complete media).

### Experimental design

Primary rabbit articular chondrocytes were distributed into 24-well plates at a concentration of 1 to 2 × 10^5 ^cells per well in 1 ml of complete media. Chondrocytes were allowed to attach for 3 to 5 days, and media were changed every 3 days. Confluent cells in multiwell plates were labeled with 1 to 2 μC/well with [^3^H]-proline during the last 24 to 48 hours of cell culture. The cell monolayer was washed at least four to five times with warm HBSS by flipping the plates to remove unincorporated proline from the matrix. Albumin- or serum-free EBSS was added to wells. Experiments were carried out in triplicate wells. The test reagents were added, and the total volume was adjusted to 0.5 ml with EBSS. The cultures were incubated at 37°C in a humidified 5% CO_2 _incubator for 4 to 24 hours. [^3^H]-proline release was measured in cell supernatant and cell lysates. A 100-μl aliquot was removed and processed for scintillation counting. The plastic-bound [^3^H]-proline-labeled matrix (that is, residuum) was solubilized with 0.5 M NaOH and counted. Percentage release of total [^3^H]-proline-labeled collagen was calculated.

### Lipoprotein and lipoprotein oxidation

The very-low-density lipoprotein and low-density lipoprotein (LDL) fractions were isolated from serum by ultracentrifugation at a density of 1.063 g/ml and were kindly provided by Vincent A. Rifici and Avedis K. Khachadurian from the Department of Medicine of our medical school [27]. Lipoproteins were tested for susceptibility for oxidation in incubation with or without glucosamine. Lipoprotein (0.25 to 0.5 mg/ml) was incubated at 30°C in phosphate-buffered saline (PBS) for 4 hours in the absence or presence of 5 μM Cu^2+ ^(copper ion) or 5 μM Cu^2+ ^and 50, 5, or 0.5 mM glucosamine. Data are expressed as malondialdehyde (thiobarbituric acid-reactive substances [TBARS]) equivalents in nanometers.

### Thiobarbituric acid-reactive substances

Two-hundred-microliter samples of TBARS that contained 50 μg of lipoprotein proteins were assayed by incubation with 1 ml of 1% thiobarbituric acid for 40 minutes at 90°C. The reaction tubes were cooled and centrifuged at 500 *g *for 10 minutes at 25°C, and the absorbencies of the supernatants were measured in a spectrophotometer at 532 nm. TBARS are expressed as nanomoles of malondialdehyde equivalents of lipoprotein protein compared with tetramethoxypropane standard [27].

### Conjugated diene formation

A washed monolayer of primary articular chondrocytes in a 60-mm Petri dish was stimulated in the presence or absence of calcium ionophore A23187 (20 μM) with or without glucosamine or vitamin E (250 μM) in phenol-free EBSS. The media were monitored for conjugated diene formation at 234 nm at different time points [28]. Delta absorbance was expressed as absorbance at different time points minus the absorbance at 0 hour. Conjugated diene in lipoproteins was determined directly by measuring the change in absorbance at 234 nm of the lipoprotein samples after incubation with Cu. Samples that contained 50 μg of protein were diluted 1:5 with PBS before measurement, and results were expressed as difference in absorbance at 234 nm.

### Preparation of cell matrix extracts

Primary articular chondrocytes in high density (1 × 10^6^/ml) were cultured in 60-mm Petri dishes to confluence, washed three times with HBSS, and set in EBSS, with or without agonist, in a total volume of 1.5 ml for variable durations. The medium and cell matrix were harvested with a cell scraper in the presence of a cocktail of protease inhibitors with EDTA (ethylenediaminetetraacetic acid), and the material was transferred to microcentrifuge tubes. One hundred fifty microliters of saturated trichloroacetic acid solution was added, and the tubes were incubated for 30 minutes on ice and microcentrifuged at 12,500 rpm for 10 minutes. The supernatants were discarded, and pellets were washed with 50 μl of ethanol and then suspended in 100 μl of sample buffer (29) and frozen at -70°C. The samples were thawed and boiled for 5 minutes with 5 μl of β-mercaptoethanol and later cooled on ice, vortexed, spun, and boiled as necessary. A total of 30 μl of each sample was loaded onto a 4% stacking gel and separated in 10% resolving SDS-PAGE gel in a mini-PROTEAN II electrophoresis cell (Bio-Rad Laboratories, Inc., Hercules, CA, USA). Electrophoresis was carried out under the reducing condition of Laemmli [29]. Proteins were stained with Coomassie Brilliant Blue.

### Immunodetection of aldehyde-protein adducts

Proteins separated by SDS-PAGE were transferred to a nitrocellulose membrane with Trans-Blot electrophoretic transfer. The blots were incubated with 50 ml of 5% bovine serum albumin (BSA) with Tris-buffered saline (TBS) (20 mM Tris/500 mM NaCl, pH 7.5) containing 0.1% Tween-20 and then were washed three times for 15 minutes with 0.5% BSA with TBS. For immunodetection, blots were incubated with antibodies diluted in 1% BSA/TBS overnight. The MDA2 mouse monoclonal antibodies, specific for malondialdehyde-modified lysine, were kindly provided by Wulf Palinski, of the University of California, San Diego (CA, USA) [30]. The monoclonal antibodies were used at dilutions of 1:2,500. The primary antibody was removed, and the blots were washed three times (15 minutes each) with TBS-containing Tween-20. The blots were then incubated in horseradish peroxidase (HRP)-labeled goat anti-mouse immunoglobulin G in 1% BSA/TBS (diluted 1:2,500) for 1 hour at room temperature. Blots were again washed with TBS (15 minutes each), and proteins were visualized as outlined in the enhanced chemiluminescence (ECL) Western blotting protocol (Amersham, now part of GE Healthcare, Little Chalfont, Buckinghamshire, UK).

### Immunodetection of protein-bound 2,4-dinitrophenylhydrazones

Derivatization with dinitrophenylhydrazones was performed as published [31]. Proteins separated by SDS-PAGE were transferred as above. For immunodetection, anti-dinitrophenyl (DNP) antibody was supplied by DAKO (Dako North America, Inc., Carpinteria, CA, USA') (V401) and used at a dilution of 1:4,000. The secondary antibody was goat anti-rabbit antibody conjugated with HRP as outlined above in the ECL Western blotting protocol (GE Healthcare).

### Statistical analysis

Results are expressed as means ± standard error of the mean. There was a 10% coefficient of variation between the mean and highest and lowest counts in random wells of each experiment. The differences of the means between groups in the same experiment were evaluated by Student *t *test (Statview^® ^program; SAS Institute Inc: Cary, NC USA). *P *values less than or equal to 0.05 were considered statistically significant.

## Results

### Glucosamine hydrochloride and glucosamine sulfate inhibit calcium ionophore-induced chondrocyte-dependent collagen degradation

**Figure 1:**
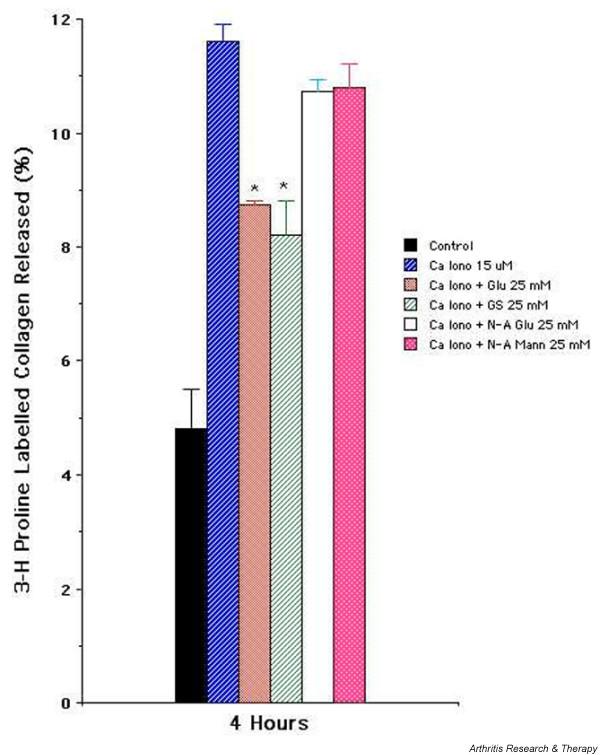
Effect of glucosamine-derived compounds on calcium ionophore-induced release of [^3^H]-proline-labeled articular collagen matrix. [^3^H]-proline-labeled monolayer of primary articular chondrocytes in 24-well plates was stimulated with calcium ionophore A23187 (15 μM) in the presence or absence of glucosamine hydrochloride (Glu) (25 mM), glucosamine sulfate (GS) (25 mM), *N*-acetyl glucosamine (N-A Glu) (25 mM), and *N*-acetyl mannosamine (N-A Mann) (25 mM). The 4-hour percentage release of labeled matrix collagen is shown. The results are presented as the mean of triplicate sets of wells ± standard error. A representative of three experiments is shown. *Statistically significant between cells stimulated with calcium ionophore and with Glu or GS. Ca Iono, calcium ionophore.

We tested the effect of glucosamine hydrochloride and glucosamine sulfate on chondrocyte-dependent collagen degradation in the previously described *in vitro *model [26]. For comparison and specificity, we also tested the effect of *N*-acetyl glucosamine and *N*-acetyl mannosamine. As shown in Figure 1, chondrocytes stimulated with calcium ionophore A23187 (15 μM) enhanced the release of [^3^H]-proline-labeled collagen as compared with the background amount of collagen released by unstimulated control chondrocytes. In the presence of 25 mM concentrations of glucosamine hydrochloride or glucosamine sulfate, there was statistically significant inhibition of the release of labeled collagen at 4 hours. In comparison, *N*-acetyl glucosamine and *N*-acetyl mannosamine did not result in inhibition of collagen degradation. The data indicate that glucosamine hydrochloride and glucosamine sulfate have specificity and significantly inhibit collagen degradation by activated chondrocytes.

### Dose and time effect of glucosamine hydrochloride and glucosamine sulfate on collagen degradation

**Figure 2:**
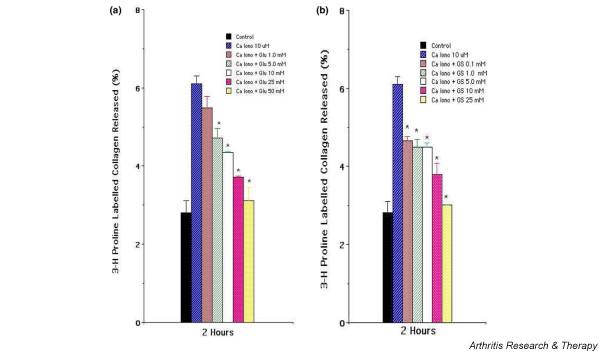
Dose-dependent effect of glucosamine hydrochloride **(a) **and glucosamine sulfate **(b) **on release of [^3^H]-proline-labeled collagen matrix by activated chondrocytes. [^3^H]-proline-labeled monolayer of primary articular chondrocytes was stimulated with A23187 (10 μM) in the absence or presence of increasing concentrations of glucosamine hydrochloride and glucosamine sulfate. The results are presented as the mean of triplicate set of wells ± standard error. A representative experiment is shown. *Statistically significant between cells stimulated with calcium ionophore and with glucosamine hydrochloride or glucosamine sulfate. Ca Iono, calcium ionophore; Glu, glucosamine hydrochloride; GS, glucosamine sulfate.

**Figure 3:**
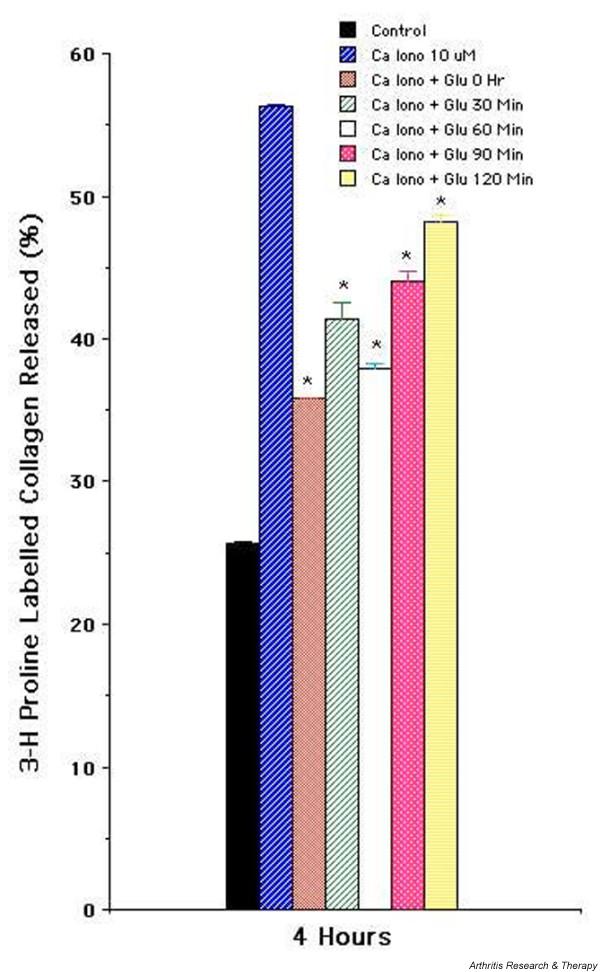
Time-dependent inhibitory effect of glucosamine hydrochloride on the release of [^3^H]-proline-labeled articular collagen matrix. [^3^H]-proline-labeled monolayer of primary articular chondrocytes was stimulated with A23187 (10 μM) in the absence and presence of glucosamine hydrochloride (50 mM). Glucosamine was added at the initiation (0 hours) or at different times as shown in the figure. The 4-hour percentage release of labeled matrix (collagen) is shown. The results are presented as the mean of triplicate set of wells ± standard error. A representative experiment is shown. *Statistically significant between cells stimulated with calcium ionophore and in the presence of glucosamine hydrochloride. Ca Iono, calcium ionophore; Glu, glucosamine hydrochloride.

As shown in Figure 2, increasing the concentration of both the glucosamine hydrochloride and glucosamine sulfate resulted in a dose-dependent inhibition of collagen degradation in calcium ionophore-stimulated chondrocyte cultures, suggesting a dose-dependent inhibitory activity on collagen degradation. Glucosamine hydrochloride (50 mM) was added at 0, 0.5, 1, 1.5, and 2 hours after stimulation of chondrocytes by calcium ionophore (10 μM) and collagen release monitored at the end of 4 hours. Addition of glucosamine hydrochloride at 0 hours resulted in significant inhibition of collagen release; a significant inhibitory effect persisted in replicate sets of cultures in which glucosamine hydrochloride was added at different time points (Figure 3). As the addition of glucosamine hydrochloride was delayed, the amount of inhibition tended to decrease but was still present. The data suggest that inhibition of collagen degradation involves downstream events of chondrocyte activation rather than interference or blockade of the early events of chondrocyte activation by calcium ionophore.

### Glucosamine hydrochloride does not inhibit conjugated diene formation by activated chondrocytes and lipoprotein oxidation

**Figure 4:**
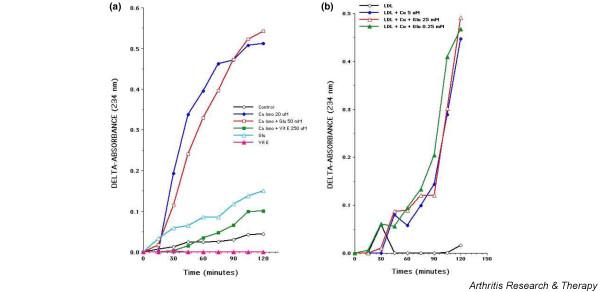
Glucosamine hydrochloride does not prevent conjugated diene formation by calcium ionophore-stimulated chondrocytes **(a) **or by copper-catalyzed oxidation of low-density lipoprotein **(b)**. **(a) **A washed monolayer of primary articular chondrocytes in 60-mm Petri dishes was stimulated in the presence or absence of A23187 (20 μm) with or without glucosamine (50 mM) or Vitamin E (250 μM) in phenol-free Earl's balanced salt solution. The media were monitored for conjugated diene formation at 234 nm at different time points. Delta absorbance shown is absorbance at different time points minus the absorbance at 0 hours. A representative of four experiments is shown. **(b) **Low-density lipoprotein (0.25 mg/ml) was incubated at 30°C in phosphate-buffered saline alone (open circles) or in the presence of 5 μM Cu^2+ ^(closed circles) or with 5 μM Cu^2+ ^and 25 mM (open triangles) or 0.25 mM (closed triangles) glucosamine. A conjugated diene formation was monitored at 234 nm. Ca Iono, calcium ionophore; Cu, copper; Glu, glucosamine; LDL, low-density lipoprotein; Vit E, vitamin E.

We monitored conjugated diene formation as an indicator of lipid peroxidation in activated chondrocytes and purified lipoprotein oxidation with or without glucosamine hydrochloride [32]. As shown in Figure 4a, calcium ionophore-stimulated chondrocytes resulted in progressive increase in the conjugated diene formation. Glucosamine hydrochloride (50 mM) did not inhibit conjugated diene formation in stimulated chondrocytes. Vitamin E (250 μM) inhibited conjugated diene formation in stimulated chondrocytes. Of note, glucosamine hydrochloride had a slight stimulatory effect on conjugated diene formation as compared with the release of conjugated diene by unstimulated control chondrocytes. There was no inhibition of conjugated diene formation in Cu-induced oxidation of purified lipoproteins by glucosamine hydrochloride (Figure 4b). Together, the data indicate that glucosamine does not inhibit initiation or progression of lipid peroxidation in chondrocytes or lipoproteins.

### Glucosamine hydrochloride inhibits TBARS formation by copper-induced lipoprotein oxidation

**Figure 5:**
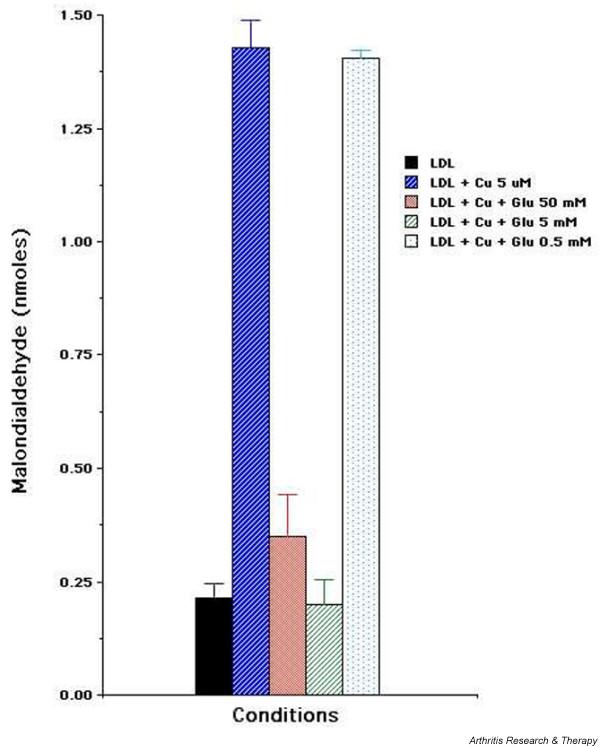
Glucosamine hydrochloride inhibits malondialdehyde formation by lipoprotein oxidation. Lipoproteins (0.5 mg/ml) were incubated at 30°C in phosphate-buffered saline for 4 hours in the absence or presence of 5 μM Cu^2+ ^or 5 μM Cu^2+ ^and 50, 5, or 0.5 mM glucosamine hydrochloride. Data are expressed as malondialdehyde equivalents in nanomoles and are presented as the mean of a duplicate set of samples ± standard error. A representative of two experiments is shown. Cu, copper; Glu, glucosamine hydrochloride; LDL, low-density lipoprotein.

We investigated the effect of glucosamine hydrochloride on TBARS formation in Cu-induced oxidation of lipoproteins. As shown in Figure 5, there was a dose-dependent inhibition of TBARS (malondialdehyde) formation by glucosamine hydrochloride. Glucosamine hydrochloride in 5 to 50 mM concentrations resulted in almost complete inhibition of TBARS formation, whereas glucosamine hydrochloride concentration of 0.5 mM had no inhibitory effect. The data suggest that glucosamine hydrochloride either interferes with the formation of downstream aldehydic products of lipid peroxidation or scavenges these products. It should be noted that glucosamine hydrochloride did not interfere in the detection of control malondialdehyde from the tetramethoxypropane standard.

### Immunoblot analysis of the effect of glucosamine hydrochloride on aldehyde-protein adduct in chondrocyte matrix extracts

**Figure 6:**
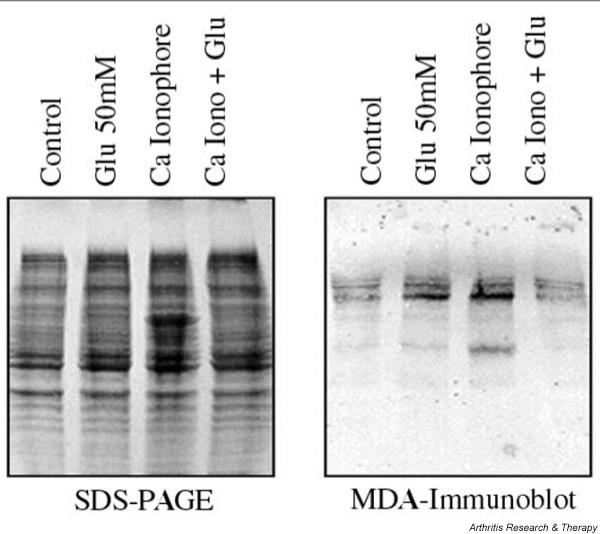
SDS-PAGE and subsequent immunoblot analysis of chondrocyte extracts. Primary confluent articular chondrocytes in 60-mm Petri dishes were washed and finally set in serum-free Earl's balanced salt solution without (control, lane 1) or with glucosamine hydrochloride (50 mM, lane 2) or with Ca Iono (20 μM, lane 3) and Ca Iono with glucosamine hydrochloride (lane 4). The chondrocytes were stimulated for 4 hours. Extracts of media-cell matrix were collected as described, and 30 μl of extracts was loaded on SDS-PAGE and transblotted onto nitrocellulose membrane. Subsequently, the membranes were reacted with MDA2 monoclonal antibodies overnight and were processed. Ca Iono, calcium ionophore; Glu, glucosamine hydrochloride.

We tested the effect of glucosamine hydrochloride on aldehyde-protein adduct formation in control and stimulated chondrocytes. Protein gel electrophoresis and immunoblot analysis using MDA2, specific for MDA-modified lysine of chondrocyte extracts, is shown in Figure 6. Extracts from control chondrocytes with glucosamine resulted in a slight increase in background immunoreactive bands to MDA2. Extracts from calcium ionophore-stimulated chondrocytes resulted in a further increase in immunoreactivity and in the appearance of new low-molecular-weight immunoreactive bands to MDA2. Increased reactivity and appearance of low-molecular-weight aldehyde-protein adducts suggest activation-dependent aldehydic protein oxidation and protein fragmentation. In comparison, extracts from calcium ionophore-stimulated chondrocyte matrix in the presence of glucosamine hydrochloride showed diminished presence and the disappearance of low-molecular-weight immunoreactive bands, suggesting that glucosamine hydrochloride diminishes aldehydic protein oxidation and fragmentation in activated chondrocyte extracts.

### Western blot analysis of effect of glucosamine on protein oxidation

**Figure 7:**
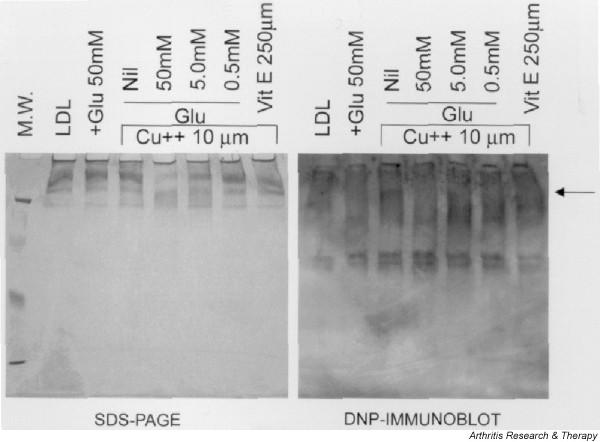
SDS-PAGE and subsequent immunoblot analysis of lipoproteins. Lipoproteins (200 μg) in a total volume of 200 μl were incubated with or without calcium (10 μM) in the absence or presence of variable concentrations of glucosamine hydrochloride for 4 hours at 30°C. The reaction was stopped by the addition of EDTA (ethylenediaminetetraacetic acid) with butylated hydroxytoluene, and aliquots were stored at -70°C. Thawed samples were derivatized with DNP, and 40 μl of sample was loaded on SDS-PAGE and transblotted onto nitrocellulose membranes. Subsequently, the membrane was incubated with anti-DNP antibodies for 1 hour and processed. DNP, dinitrophenyl; Glu, glucosamine hydrochloride; LDL, low-density lipoprotein; M.W., molecular weight; Vit E, vitamin E. Arrow indicates diffused DNP reactivity to high molecular weight lipoproteins in Lane 3.

We tested the effect of glucosamine hydrochloride on lipoprotein protein oxidation using the identification of protein carbonyls as one of the modifications as described in oxidized proteins [33,34]. The carbonyl groups generated on oxidized proteins were allowed to react with 2,4-dinitrophenylhydrazine and this group is recognized by anti-DNP antibodies [31]. As shown in the DNP immunoblot in Figure 7, the addition of glucosamine hydrochloride alone did not generate carbonyl modification in lipoproteins as compared with control. Lipoproteins oxidized with Cu resulted in diffused DNP immunoreactivity to high-molecular-weight lipoproteins (as indicated by the arrow in lane 3). This diffused DNP immunoreactivity was obliterated by a 50 mM concentration of glucosamine hydrochloride in Cu-oxidized lipoproteins (as seen in lane 4), suggesting that glucosamine prevents formation of carbonyl groups in oxidized proteins. On the other hand, glucosamine hydrochloride in concentrations of 5.0 mM or 0.5 mM had little effect on DNP immunoreactivity (as seen in lanes 5 and 6). Two bands of low-molecular-weight DNP immunoreactive bands were observed in control and Cu-stimulated lipoproteins, and glucosamine had no discernable effect on their signal intensity.

## Discussion

Using this *in vitro *model of chondrocyte activation-dependent collagen degradation, we show that glucosamine specifically and significantly inhibited collagen degradation. Inhibition of collagen degradation by glucosamine was not mediated by inhibiting the chondrocyte lipid peroxidation process but by inhibiting advanced lipoxidation reactions. Specifically, glucosamine inhibited purified lipoprotein protein oxidation and aldehydic oxidation of chondrocyte matrix.

Using this *in vitro *model, we had previously shown [26,35,36] that chondrocyte-derived lipid radicals specifically mediate degradation of cartilage collagen [26,35]. This model therefore is a fair representation of cartilage collagen degradation. The relevance of this *in vitro *model to human OA pathogenesis was demonstrated by detection of *in vivo *molecular imprints of lipid peroxidation in which OA and normal cartilage tissue sections were studied [36]. We also demonstrated the presence of OA disease-specific malondialdehyde and hydroxynonenal adducts in human OA cartilage tissue sections, suggesting the *in vivo *role of lipid peroxidation in the OA pathogenesis [36,37]. Collectively, these observations indicate that lipid peroxidation may play a larger role in the pathogenesis OA than has previously been recognized.

We investigated the effect of glucosamine in our assay system. As shown, only glucosamine hydrochloride or glucosamine sulfate specifically and significantly inhibited collagen degradation by activated chondrocytes and the effect was dose-dependent. Similar effects by both agents (glucosamine hydrochloride and glucosamine sulfate) excluded the possibility that the inhibition observed was mediated by the sulfate moiety in the latter compound. Glucosamine hydrochloride had little or variable effect on hydrogen peroxide-induced collagen degradation, suggesting that it did not inhibit oxygen radical/hydrogen peroxide-mediated collagen degradation (data not shown).

Since the mechanism of collagen degradation in this model appears to involve the activation of lipid peroxidation in chondrocytes, it raises the possibility that glucosamine was acting like a chain-breaking antioxidant similar to vitamin E. However, glucosamine had no discernable effect on conjugated diene formation by activated chondrocytes, suggesting that its mechanism of action was not due to chain-breaking antioxidant activity. As expected, vitamin E inhibited conjugated diene formation by chondrocytes. To further confirm these findings, we tested the effect of glucosamine in a purified lipoprotein oxidation model system, a commonly used *in vitro *model for studies on lipoxidative modification of proteins [27]. Again, glucosamine hydrochloride had no discernable effect on Cu-induced conjugated diene formation in lipoproteins. Furthermore, glucosamine did not cause an increase in the lag phase of LDL oxidation or a decrease in absorbance at 234 nm during the later plateau phase of the reaction. Together, these observations indicate that glucosamine does not interfere with initiation or propagation of lipid peroxidation reaction.

The inhibition of collagen degradation by glucosamine was manifested even when the addition of glucosamine was delayed in activated chondrocyte cultures, indicating that its mechanism of action involved downstream events of chondrocyte activation rather than interfering with or blocking the early events of chondrocyte activation by calcium ionophore. We tested the effect of glucosamine on TBARS formation by Cu-induced oxidation of purified lipoproteins. Glucosamine in a dose-dependent manner inhibited malondialdehyde formation by oxidized lipoprotein. The data suggest that glucosamine either inhibited or scavenged aldehydic products of lipid peroxidation. However, glucosamine did not interfere in the detection of control malondialdehyde in TBARS assay, suggesting that most likely glucosamine inhibited advanced lipoxidation reactions rather than scavenging aldehydic products.

The identification of aldehydic adducts provides a molecular clue of chondrocyte matrix damage mediated by lipid-free radicals [26]. On immunoblot analysis of the effect of glucosamine, we identified activation-dependent low-molecular-weight MDA adduct in chondrocyte matrix extracts; the intensity of higher-molecular-weight aldehydic adducts increased in activated chondrocyte extracts as compared with extracts from control chondrocyte matrix. In the presence of glucosamine, the low-molecular-weight aldehydic adducts in activated extracts disappeared whereas the intensity of high-molecular-weight adducts decreased, indicating that glucosamine prevented oxidation and/or fragmentation of chondrocyte matrix components. These observations are consistent with the finding that glucosamine inhibited malondialdehyde (TBARS) formation in Cu-induced oxidation of lipoprotein. Together, these observations suggest that glucosamine inhibits advanced lipoxidation reactions. By preventing advanced lipid-free radical production, glucosamine perhaps inhibits collagen degradation observed in the *in vitro *model system.

Inhibitors of advanced lipoxidation reactions such as aminoguanidine and pyridoxamine have been evaluated in animal models of diseases such as diabetes [38,39]. These compounds are being evaluated in clinical trials for the treatment of diabetic nephropathy [40]. Aminoguanidine inhibits chemical modification of proteins during lipid peroxidation reactions and inhibits metal-catalyzed oxidation of LDLs and uptake of oxidized LDL into macrophages via the scavenger receptor [41,42]. Pyridoxamine has also been shown to have potent advanced lipoxidation inhibitory activities in a variety of tests [38,39]. In addition to showing advanced lipoxidation inhibitory activity, these compounds show inhibitory activity against advanced glycation reactions (AGEs) [38,43]. AGE products formed during autoxidation of carbohydrates and lipid peroxidation reactions produce reactive carbonyl species that cause a carbonyl modification reaction in protein structure and function and cause the formation of high-molecular-weight protein aggregates [33]. Osteoarthritic cartilage shows increased levels of insoluble protein aggregates and AGE-modified products [44-46]. Identification of carbonyl modification of proteins provides a powerful tool to monitor the development of a number of pathologies mediated by a condition commonly described as 'carbonyl stress' [33,34,47]. As shown, glucosamine inhibited Cu-induced carbonyl modification of lipoproteins, indicating that glucosamine also traps reactive carbonyl compounds. In addition to aminoguanidine and pyridoxamine, therapeutic agents such as L-arginine, OPB-9195, tenilsetam, and metformin have been proposed to trap reactive carbonyl compounds [48-53].

The pharmacokinetics of oral administration of glucosamine sulfate show that plasma levels increase more than 30-fold from baseline and peak at approximately 10 μM with the standard 1,500-mg once-daily dosage [54]. We postulate that because *in vivo *tissue levels of glycosaminoglycans in cartilage are hundreds perhaps thousands of folds higher than in serum or joint fluids, glucosamine, which is a structural component of aggrecan, may locally provide an antioxidant environment that may protect cartilage collagen from oxidative damage.

Our data suggest that the decrease in collagen degradation by glucosamine observed in this *in vitro *model system may be mediated by the inhibition of advanced lipoxidation reaction, preventing the oxidation and loss of collagen matrix from labeled chondrocyte matrix. Further studies are needed to relate these *in vitro *findings to the retardation of cartilage degradation reported in OA trials investigating glucosamine.

## Conclusion

In an *in vitro *model of cartilage collagen degradation in which collagen degradation induced by activated chondrocytes is mediated by lipid peroxidation reaction, glucosamine decreases collagen degradation by inhibiting advanced lipoxidation reaction and thus prevents the oxidation and loss of collagen matrix from labeled chondrocyte matrix.

## Abbreviations

AGE = advanced glycation reaction; BSA = bovine serum albumin; Cu = copper; DMEM = Dulbecco's modified Eagle's medium; DNP = dinitrophenyl; EBSS = Earl's balanced salt solution; ECL = enhanced chemiluminescence; FBS = fetal bovine serum; HBSS = Hanks' balanced salt solution; HRP = horseradish peroxidase; IL-1 = interleukin-1; LDL = low-density lipoprotein; OA = osteoarthritis; PBS = phosphate-buffered saline; TBARS = thiobarbituric acid-reactive substances; TBS = Tris-buffered saline.

## Competing interests

The authors declare that they have no competing interests.

## Authors' contributions

MLT developed the study experimental protocol. All authors participated in conducting and analyzing the experiments. All authors were involved in the drafting, review, and final approval of the manuscript.
